# Studies on the Acidities of C_2_ Symmetrical Chiral Phosphoric Acids Based on a Decahydroquinoxaline Scaffold

**DOI:** 10.1021/acsomega.5c13291

**Published:** 2026-06-17

**Authors:** Margherita Gazzotti, Daniele Fumagalli, Valentina Pifferi, Laura Raimondi, Sergio Rossi

**Affiliations:** Dipartimento di Chimica, 9304 Università Degli Studi di Milano, via Golgi 19, Milano 20133, Italy

## Abstract

The p*K*_a_ values of 34 new C_2_-symmetrical chiral phosphoric acids based on a decahydroquinoxaline scaffold were computed by DFT calculations. The p*K*_a_ values were computed in DMSO and acetonitrile using two computational methods: the isodesmic approach and the linear free energy scaling relationship (LFESR) approach. Both methods were first benchmarked against experimental data for BINOL-derived chiral phosphoric acids and then applied to a wide series of decahydroquinoxaline-based chiral phosphoric acids, highlighting trends in acidity correlated to the electronic nature of substituents on the decahydroquinoxaline scaffold. Experimental validation by potentiometric titration confirmed that the predicted p*K*_a_ values are in good agreement with measurements, with differences generally below 1 p*K*_a_ unit. These results provide a deeper understanding of the relationship between structure and acidity in CPAs and represent a simulating model for the rational design of next-generation chiral phosphoric acid catalysts in stereoselective transformations.

## Graphical abstract

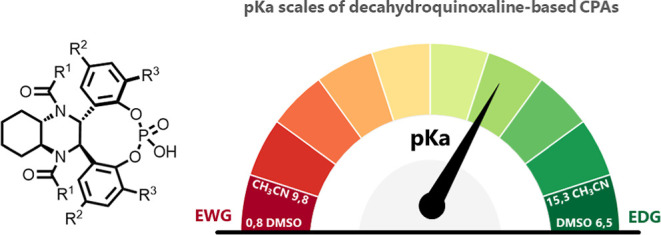

## 1 Introduction

Chiral Phosphoric Acids (CPAs) have found extensive application as organocatalysts in stereoselective synthesis, thanks to their ability to promote a broad range of enantioselective transformations under mild reaction conditions.^1–5^ Their catalytic activity arises from the simultaneous presence of a Brønsted acid site (OH group) and a Lewis basic site (P = O moiety), which usually are both involved in the activation of substrates according to a bifunctional activation pathway.

Following Akiyama and Terada pioneering work with BINOL-derived CPAs,^6,7^ many different chiral scaffolds have been investigated over the past two decades to expand the structural diversity of this class of compounds. In this context, we have recently introduced a novel class of C_2_ symmetrical chiral phosphoric acids featuring a chiral decahydroquinoxaline backbone, which have proven to be highly effective in the Friedel–Crafts alkylation of indoles with *N*-tosylimines.^8^ Differences in reactivity among these catalysts are usually correlated with both the steric hindrance and the electronic nature of the substituents (presence of electron-withdrawing or electron-donating groups on the chiral scaffold), which are presumed to modulate their acidity.^9^ However, a systematic evaluation of their Brønsted acidity, expressed in terms of p*K*_a_, remains largely unexplored. This lack of data is quite surprising, since p*K*_a_ is a fundamental parameter that regulates the nature of catalyst–substrate interactions and, by extension, provides a basis for rationalizing the chemical efficiency and selectivity of a given CPA in specific stereoselective transformations. Although acid-base reactivity plays a central role in driving many chemical transformations, its study is quite challenging due to the difficulty in estimating solvent interactions during the ion solvation process. Typically, p*K*_a_ values are determined for only a few representative compounds,^10–12^ and even in these cases, the reported values can be significantly different depending on the experimental method or solvent used for their determination.^13,14^

In response to the limited availability of experimental p*K*_a_ data, and to overcome some of these problems, different computational approaches have been developed.^15^ Nonetheless, these methods also present limitations, as their reliability depends on the theoretical assumptions adopted, factors that become particularly critical in nonaqueous solvents.^16–19^ As a result, there is a concrete risk of drawing misleading conclusions if such computational predictions are not validated or not critically assessed. Quantum chemical methods have been widely employed for p*K*_a_ predictions,^20^ using either direct methods^21,22^ or undirect ones (in that case, commonly referred to as the “isodesmic method”).^23–27^ As alternatives, machine learning approaches,^28^ metadynamics simulations,^29,30^ as well as computational tools based on descriptors have been also developed.^31–33^

Direct methods are based on Born–Haber cycles,^20^ and results obtained with this approach closely align with experimental measurements. However, this high level of accuracy requires high computational costs, especially when an explicit solvation model is used, making the approach resource-intensive and sometimes impractical for larger systems. On the other hand, undirect methods are less demanding in terms of computational resources since they relate the target reaction to a reference dissociation reaction with a known p*K*_a_ value. Clearly, as a drawback, this approach suffers from the availability of experimental p*K*_a_ data to be used as reference compounds, which are not always available.

This computational strategy was employed in 2013 by Cheng, Li, and co-workers to estimate the acidity in DMSO of over 40 chiral phosphoric acids based on the BINOL scaffold.^34^ The p*K*_a_ values were predicted by fully optimizing the CPA structures at the B3LYP/6–31 + G­(d) level of theory, while the standard free energies of solvation in DMSO were computed using the SDM model at the M06–2X/6–311++G­(2df,2p) level. According to this approach, the predicted p*K*_a_ values for known reference systems showed a deviation at least of ±0.4 p*K*_a_ units from the corresponding experimental values obtained by spectrophotometric methods.^35^

More recently, in 2022, Busch and co-workers reported a more general computational method to predict p*K*_a_ values of any compounds across different solvents,^36^ based on experimental aqueous p*K*_a_ values of reference compounds combined with absolute potentials of the standard hydrogen electrode (SHE) in nonaqueous media.^37^

Inspired by these developments, we applied both the isodesmic method and the linear free energy scaling relationship (LFESR) approach to estimate the p*K*_a_ values of newly developed chiral phosphoric acids based on the decahydroquinoxaline scaffold,^8^ evaluating acidities in both DMSO and CH_3_CN solvents.

## 2 Results and Discussion

To gain deeper insight into the acid-base properties of the newly developed CPA series, both the isodesmic method and the LFESR were investigated. p*K*_a_ values obtained with this evaluation will be helpful in rationalizing their reactivity across different chemical transformations. It has been demonstrated that stronger chiral phosphoric acids (p*K*_a_ < 2 in DMSO) not only efficiently promote classical imine activations but are also capable of facilitating more challenging C–C bond activations.^38^ The computed acidity trends could then guide the rational design of CPAs with optimized acidities for targeted stereoselective reactions.

### 2.1 p*K*_a_ Values Computed According to the Isodesmic Method

In computational approaches, the acidity of a compound is typically assessed through the Gibbs free energy change associated with its acid dissociation reaction, which is directly related to the p*K*_a_ value. The dissociation process for a weak acid in a given solvent is described by eq 1:

1 $${{HA}_{\left(solv\right)\overset{\Delta Gsolv}{⇌}}}_{H_{\left(solv\right)}^{⊕}+A_{\left(solv\right)}^{⊖}}$$

and the p*K*_a_ associated with this process is calculated as

2 $${pK}_{a}=\frac{{-\Delta G}_{\left(solv\right)}^{*}}{RT\ln\left(10\right)}$$

where Δ*G*_(solv)_^*^ represents the standard Gibbs free energy change of the dissociation in solution, *R* is the universal gas constant, *T* is the temperature in Kelvin, and the superscript * indicates standard-state conditions (1 mol·L^–1^ at 298.15 K). The value of Δ*G*_(solv)_^*^ can, in turn, be derived from

3 $${\Delta G}_{\left(solv\right)}^{*}=G^{*}\left(A_{\left(solv\right)}^{-}\right)+G^{*}\left(H_{\left(solv\right)}^{+}\right)-G^{*}\left({HA}_{\left(solv\right)}\right)$$

where *G* ^*^(A^–^_(solv)_), *G* ^*^(H_(solv)_^+^), and *G* ^*^(HA_(solv)_) represent the standard-state absolute Gibbs free energies of the deprotonated and protonated forms of the acid and of the acid itself, respectively. Since H^+^ does not exist as a free species but interacts strongly with the solvent (by hydrogen bonding or by dipolar interactions), its absolute free energy is not measured directly. Instead, it is usually estimated indirectly using thermodynamic cycles that involve an exchange proton between two acids: a reference acid with a known p*K*_a_ (Scheme 1, highlighted in pink) and the target acid whose p*K*_a_ is to be computed (highlighted in blue).

**Scheme 1:**
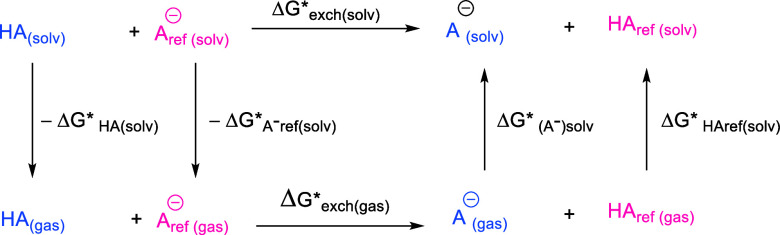
Thermodynamic Cycle Used to Compute the Standard Free Energy Change for a Chiral Phosphoric Acid in DMSO and CH_3_CN According to the Proton-Exchange Method^a^

a The acid with an experimentally determined p*K*_a_ is shown in pink, while the target acid, whose p*K*_a_ needs to be computed, is highlighted in blue.

This thermodynamic cycle avoids to directly calculate the proton dissociation free energy by combining gas-phase contributions and solvation energies of both the neutral acids (HA) and their conjugate bases, but it is important that the structure of the reference acid closely resembles the geometries of acids which are under investigation in order to reduce the risk of significant inaccuracies. According to Scheme 1, vertical arrows represent solvation processes, each associated with a solvation free energy, while the horizontal arrows correspond to acid dissociation in the gas phase (Δ*G*_exch(gas)_^*^) and in the solvent of choice (Δ*G*_exch(solv)_^*^). Based on this thermodynamic cycle, the free energy change for the dissociation of a generic acid HA in a desired solvent can be rewritten as

4 $${\Delta G}_{exch\left(solv\right)}^{*}={\Delta G}_{exch\left(gas\right)}^{*}+{\Delta G}^{*}\left(A_{\left(solv\right)}^{-}\right)+{\Delta G}^{*}\left({HA}_{ref\left(solv\right)}\right)-{\Delta G}^{*}\left({HA}_{\left(solv\right)}\right)-{\Delta G}^{*}\left(A_{ref\left(solv\right)}^{-}\right)$$

and p*K*_a_ can be calculated as

5 $${pK}_{a}=\frac{{-\Delta G}_{exch\left(solv\right)}^{*}}{RT\ln\left(10\right)}+{pK}_{a}\left({HA}_{ref\left(solv\right)}\right)$$

Since p*K*_a_ values of some BINOL-derived chiral phosphoric acids have been already experimentally measured in DMSO or in CH_3_CN, these can serve as reliable reference values for the prediction of p*K*_a_ of unknown CPA derivatives in the same solvent. By calculating the corresponding standard free energy change of acid dissociation in solution Δ*G*_exch(solv)_^*^of a target derivative, it becomes possible to estimate its p*K*_a_ value with good accuracy.

The selection of an appropriate reference compound is a critical aspect of the isodesmic approach since the accuracy of relative p*K*_a_ calculations strongly depends on the structural similarity between the reference acid and the target systems. In our study, we used the p*K*_a_ values measured in DMSO by Berkessel and co-workers as reference data, as their work offers a consistent and coherent data set obtained using the same experimental method across a series of chiral phosphoric acids.^34^ This choice was made to reduce variability and avoid potential discrepancies that could arise from comparing p*K*_a_ values reported by different authors using different experimental protocols. For CH_3_CN, the reference p*K*_a_ values reported by Rueping and Leito were used.^40^

A slight modification of the protocol reported by Cheng and Li was adopted. Initial conformational geometries were obtained through Monte Carlo conformational analysis performed with Molecular Mechanics calculations using the OPLS2005 force field^41^ of the Macromodel package in the Schrodinger suite.^42^ For molecules exhibiting multiple conformers, the structure within 3 kcal/mol was fully optimized by DFT calculations in the gas phase using the M06–2X functional^43^ with the 6–31 + G­(d) basis set implemented in the Gaussian package.^44^ Harmonic vibrational frequency calculations were performed at the same level of theory to confirm the nature of the stationary points and to obtain thermal corrections to the Gibbs free energy, and the structure with the lowest free Gibbs energy was selected for subsequent calculations. The M06–2X functional was chosen for its improved performance in describing long-range dispersion interactions compared to the B3LYP functional.^45^ Single-point energy calculations were subsequently carried out on the optimized structures at the M06–2X/6–311++G­(2df,2p) level of theory, including solvent effects via the SMD solvation model^46^ for DMSO and CH_3_CN. The previously calculated thermal corrections were then added to the electronic energies to obtain the final Gibbs free energies used for the p*K*_a_ determination. The conjugate bases of each acid were generated by removing a proton from the corresponding acid structure. No conformational analysis was performed for these species; geometries were directly reoptimized by using the same DFT methodology described above.

BINOL-derived CPA **1** with a p*K*_a_ of 3.37 in DMSO was used as the reference structure for calculations in this solvent,^34^ whereas compound **2** with a p*K*_a_ of 12.73 in CH_3_CN was selected as the reference for calculations in acetonitrile (Scheme 2).^39^

**Scheme 2:**
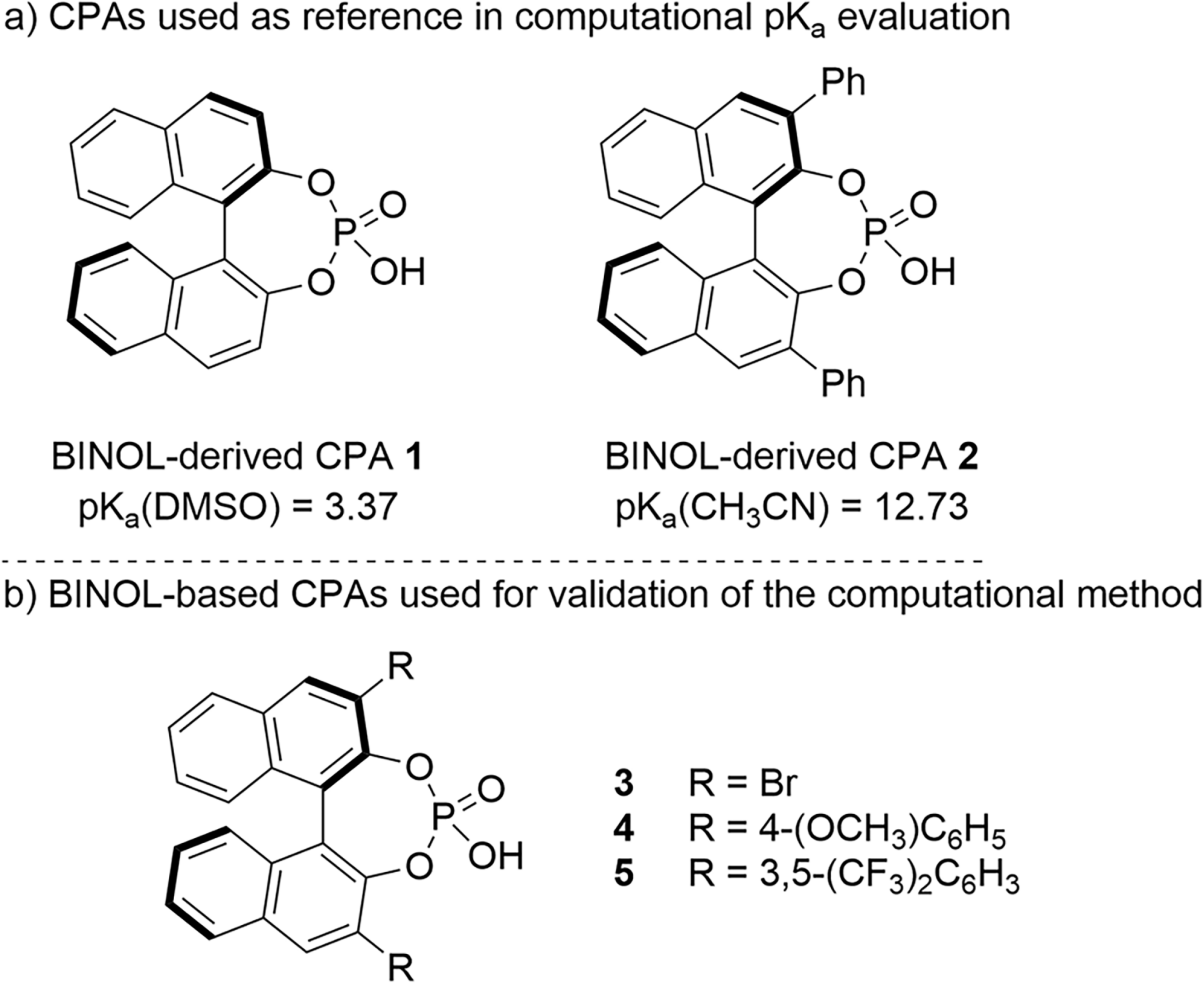
(a) Reference BINOL-Derived Chiral Phosphoric Acids; (b) BINOL-Based Chiral Phosphoric Acids Investigated through p*K*_a_ Calculations

CPAs **3–5**, for which experimental p*K*_a_ values are available in DMSO, were included as a validation set for the computational protocol. The results are reported in Table 1.

**Table 1:** p*K*_a_ Values of Various BINOL-Based Chiral Phosphoric Acids Calculated Using the Proton-Exchange Method in Different Solvents

entry	CPAs	p*K*_a(exp)_ ^a^	p*K*_a(calc)_ DMSO^b^	p*K*_a(calc)_ DMSO^c^	p*K*_a(calc)_ CH_3_CN^c^
1	**1**	3.37	-	-	12.09
2	**2**	3.86	3.55	3.85	-
3	**3**	2.90	2.33	2.42	10.77
4	**4**	3.49	3.69	4.05	12.81
5	**5**	2.63	2.85	2.79	11.79

a Determined in DMSO by Berkessel, O’Donoghue, and co-workers using spectrophotometric methods; see ref 35.

b Calculated values at SMD­(DMSO)/M06–2X/6–311++G­(2df,2p)//B3LYP/6–31G+(d) by Chen, Li, and co-workers; see ref 34.

c Calculated by SMD/M06–2X/6–311++G­(2df,2p)//M06–2X/6–31G+(d).

The data show that switching the geometry optimization functional from B3LYP to M06–2X leads to better agreement with experimental p*K*_a_ values in DMSO, with M06–2X providing more accurate predictions, particularly for compounds **2** and **5**. Although no experimental p*K*_a_ data are available in acetonitrile for direct comparison, the calculated acidity trends in this solvent are consistent with the behavior shown from DMSO, supporting the reliability of the computational approach.

Given the slightly superior performance of M06–2X in modeling acid–base equilibria in DMSO and the tendency of B3LYP to slightly underestimate p*K*_a_ values,^35^ M06–2X was chosen for all subsequent calculations. This choice is further supported by the lower mean unsigned error (MUE) obtained with M06–2X (0.30) compared to that with B3LYP (0.33), confirming its better overall agreement with experimental data.

Next, we applied this computational protocol to estimate the p*K*_a_ values of a new series of chiral phosphoric acids derived from a decahydroquinoxaline scaffold. The p*K*_a_ values of compounds **6–14**, previously demonstrated to be effective organocatalysts in the enantioselective Friedel–Crafts alkylation of indoles with *N*-tosylimines,^8^ were subsequently investigated in both DMSO and acetonitrile. The results are summarized in Scheme 3.

**Scheme 3:**
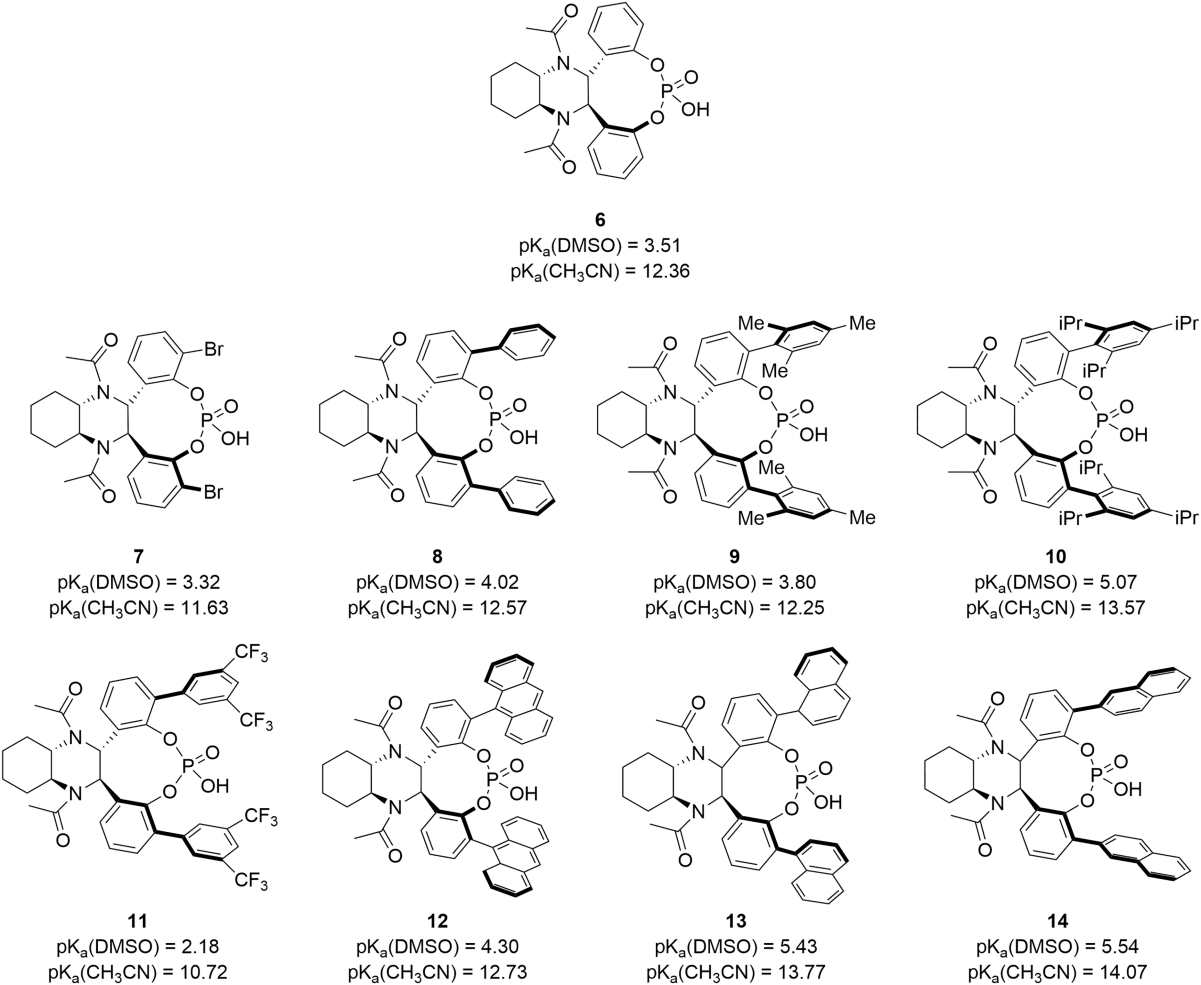
Computed p*K*_a_ Values of Chiral Phosphoric Acids Based on the Decahydroquinoxaline Scaffold in DMSO and Acetonitrile Calculated at the SMD/M06-2X/6-311++G­(2df,2p)//M06-2X/6-31G+(d) Level of Theory

As expected, compound **11**, bearing two 3,5-trifluoromethylphenyl substituents in *ortho* positions to the acidic function, shows the lowest p*K*_a_ value (2.18 in DMSO). At the same time, CPA **7**, functionalized with two bromine atoms, also exhibits a relatively low value of p*K*_a_. This fact is consistent with the known effect of EWG substituents to increase acidity by stabilizing the conjugate base through an –I inductive effect. On the other hand, compounds **9** and **10**, bearing electron-donating substituents, and compounds **13** and **14**, showing extended conjugation through aromatic double bonds, are characterized by higher p*K*_a_ values, since these substituent patterns could destabilize the conjugate base by the +I effect. Notably, compound **14**, featuring bulky, conjugated aryl substituents, has a p*K*_a_(DMSO) = 5.54. By contrast, the higher acidity of compound **9** compared to that of **8**, as well as the acidity of **12** compared with CPAs **13** and **14**, represents an exception to this general trend, suggesting that the observed acidity pattern is not governed exclusively by electronic effects and that steric and conformational factors must also be taken into account.

A similar trend was observed for the p*K*_a_ values computed in acetonitrile. In this solvent, the p*K*_a_ values are consistently higher than those obtained in DMSO, which can be attributed to the lower basicity and reduced anion-stabilizing ability of acetonitrile. Among p*K*_a_ values computed in CH_3_CN, compound **11** still shows the highest acidic character with a p*K*_a_(CH_3_CN) = 10.72, whereas chiral phosphoric acid **14** remains the least acidic one (p*K*_a_(CH_3_CN) = 14.07).

Since the decahydroquinoxaline scaffold offers multiple sites of functionalization easily accessible from a synthetic point of view,^8^ we explored the acidic properties of a series of decahydroquinoxaline-based chiral phosphoric acids **15–34** bearing structural modifications at R^1^, R^2^, and R^3^ positions. The p*K*_a_ values were computed in both DMSO and CH_3_CN as solvents, and the results are reported in Table 2.

**Table 2:**
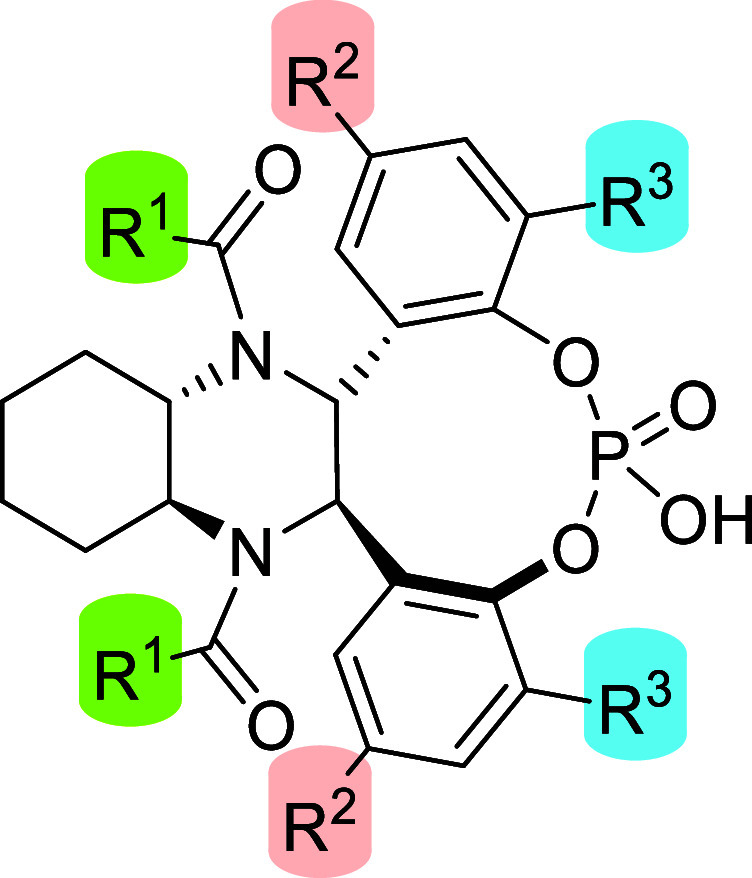
Computed p*K*_a_ Values in DMSO and CH_3_CN for Chiral Phosphoric Acids Performed at the SMD/M06-2X/6-311++G­(2df,2p)//M06-2X/6-31G+(d) Level of Theory

entry	R^1^	R^2^	R^3^	CPAs	p*K*_a(calc)_ DMSO	p*K*_a(calc)_ CH_3_CN
1	CH_3_	H	C_6_F_5_	**15**	0.85	9.84
2	CH_3_	H	cyclohexyl	**16**	3.11	12.63
3	CH_3_	H	4-(CH_2_–CH–CH_2_)C_6_H_4_	**17**	4.06	12.83
4	CH_3_	H	4-(OCH_3_)C_6_H_4_	**18**	4.39	12.91
5	CH_3_	H	4-(CH_3_–CH=CH)C_6_H_4_	**19**	5.30	13.84
6	CH_3_	CH_3_	Ph	**20**	5.14	13.57
7	CH_3_	CH_3_	3,5-(CF_3_)_2_–C_6_H_3_	**21**	3.23	12.09
8	H	H	H	**22**	3.37	12.19
9	H	H	Ph	**23**	4.68	13.36
10	H	H	2,4,6-(*i*Pr)_3_-C_6_H_2_	**24**	5.61	13.92
11	Ph	H	H	**25**	3.27	11.78
12	Ph	H	Ph	**26**	3.81	12.49
13	*i*Pr	H	H	**27**	2.98	11.60
14	2,4,6-(CH_3_)_3_C_6_H_2_	H	H	**28**	3.57	11.69
15	2,4,6-(*i*Pr)_3_C_6_H_2_	H	H	**29**	3.71	12.01
16	3,5-(CF_3_)_2_–C_6_H_3_	H	H	**30**	2.82	11.57
17	CF_3_	H	H	**31**	2.02	10.96
18	C_6_F_5_	H	H	**32**	2.12	10.95
19	1-naphthyl	H	H	**33**	6.53	15.29
20	2-naphthyl	H	H	**34**	4.48	12.71

CPA **15** (entry 1), bearing a pentafluorophenyl group at the R^3^ position, exhibits the highest acidity among all the structures investigated so far with a p*K*_a_(DMSO) = 0.85. In contrast, compounds **16**–**19**, in which the R^3^ substituent is a cyclohexyl, a 4-allyl-phenyl, a *p*-methoxyphenyl, or a 4-(CH_3_–CH=CH)­C_6_H_4_ group, show progressively higher p*K*_a_ values, ranging from 3.11 to 5.30 as a consequence of the increased electron-donating character of the substituents (entries 2–4). Compounds **20** and **21**, bearing a methyl group on the *para* position of the phenol ring, exhibit a reduced acidic character compared to their analogues **8** and **11**. This is reflected in an increase in p*K*_a_ values of approximately one unit (5.14 *vs* 4.02 for compound **20** *vs* compound **8** and 3.23 *vs* 2.18 for compounds **21** *vs* **11**). This observation suggests that despite being in the *para* position relative to the phosphoric acid moiety, a methyl substituent can still exert an electronic influence on the overall acidity of the catalyst.

We then turned our attention to the modification of the amide moiety by evaluating the different R^1^ substituents. The replacement of an acetyl group with a formyl group leads to a slight increment of acidity in compound **22** compared to its analogue **6**. However, compounds **23** and **24** showed only modest changes in p*K*_a_ values relative to their acetyl analogues **8** and **10** and were actually less acidic. Additionally, compound **25** bearing a benzoyl group exhibited a modest decrease in p*K*_a_ compared to CPA **8** (3.27 *vs* 4.02 in DMSO) but showed no significant difference when compared to compound **22**. These data indicate that the modification of the amide moiety leads to only minor perturbations in the acidity.

CPA **26**, characterized by the presence of a benzoic moiety at the nitrogen atom (entry 12), exhibited a p*K*_a_ of 3.81 in DMSO, which is slightly lower than that of its analogues bearing a *N*-formyl (compound **23**) or *N*-acetyl substituent (compound **8**) but less acidic than compound **25**, where the absence of a phenyl group at the R^3^ position (entry 11 *vs* 12) results in a 0.5 unit decrease in p*K*_a_, making compound **25** more acidic than compound **26**. The introduction of more electron-donating groups such as mesityl (entry 14) and triisopropylphenyl (entry 15) also led to compounds **28** and **29** with higher p*K*_a_ values (3.57 and 3.71, respectively) compared to compound **11**.

Compound **30** (entry 16), bearing the moderately electron-withdrawing 3,5-bis­(trifluoromethyl)­phenyl group, exhibits a p*K*_a_ of 2.82 in DMSO comparable to compounds with bulky electron-donating groups such as compound **27**. In contrast, the presence of stronger electron-withdrawing substituents results in a marked increase in acidity; compounds **31** and **32** bearing CF_3_ (entry 17) and pentafluorophenyl (entry 18) groups show p*K*_a_ values of 2.02 and 2.12, respectively.

The presence of aromatic systems such as in 1-naphthyl derivative **33** (entry 19) shows a p*K*_a_(DMSO) of 6.53, while the 2-naphthyl analogue **34** is more acidic (entry 20, p*K*_a_ = 4.48), suggesting that the presence of extended aromatic conjugation significantly affects the electronic distribution and, consequently, the acidity of the final compound.

Despite differences in polarity and hydrogen-bond-acceptor ability between DMSO and CH_3_CN, the computed p*K*_a_ values in acetonitrile exhibit a similar trend compared to those obtained in DMSO. The most acidic compound is catalyst **15**, bearing a pentafluorophenyl substituent close to the phosphoric acid moiety (p*K*_a_(CH_3_CN) = 9.84), followed by compounds **31** and **32**, featuring electron-withdrawing groups on the amide functionality. By contrast, the least acidic CPA is compound **33** (R^1^ = 1-naphthyl), which shows the highest p*K*_a_ value in both solvents.

Overall, the computed p*K*_a_s in both DMSO and CH_3_CN confirm that the acidity of CPAs based on a decahydroquinoxaline scaffold is primarily dictated by the electronic nature of the R^3^ substituents, whereas modifications to the substituents on the amide groups play a secondary but significant role.

### 2.2 p*K*_a_ Values Computed Using the LFESR Approach

The isodesmic method enabled the calculation of p*K*_a_ values for CPAs **6–34**; however, its requirement for a reference acid with both a known experimental p*K*_a_ and closely related structural features limits its broader applicability. In this study, BINOL-derived CPAs **1** and **2** were employed as reference acids; nevertheless, their structural and electronic characteristics may not be fully compatible with those of the decahydroquinoxaline-based CPAs, which could lead to systematic deviations in the p*K*_a_-computed values.

To address this issue, we investigate the linear free energy solvation relationship (LFESR) approach recently developed by Busch and co-workers for the estimation of acid p*K*_a_. This methodology provides an alternative strategy for predicting p*K*_a_ values across a wide range of solvents without requiring experimental p*K*_a_ data in the solvent of interest, since the water dissociation of formic acid to formate and a proton was selected as a universal reference reaction (p*K*_a_(H_2_O) = 3.77).^36^ According to this approach, the dissociation process of a desired compound is modeled via a Born–Haber cycle which involves a proton-coupled electron-transfer oxidation (PCET step) followed by an electron-transfer reduction (ET step), as shown in Scheme 4.

**Scheme 4:**
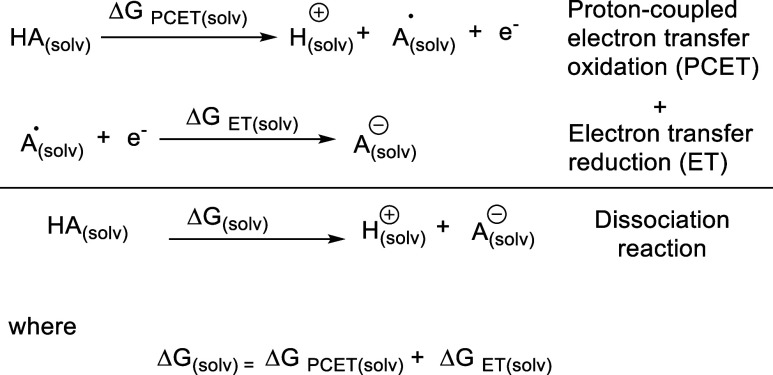
Thermochemical Cycle Connecting the p*K*_a_ with Proton-Coupled Electron-Transfer and Electron-Transfer Reactions

As a result, the overall Gibbs free energy associated to the dissociation process can be calculated as the sum of the Gibbs free energy of these two separate processes. This approach allows the ET step to be directly correlated with the absolute potential of the standard hydrogen electrode (SHE) in water. Since the proton-coupled electron-transfer (PCET) step needs to be computed in the solvent of interest, whereas the ET step is referenced to water, a solvent correction factor must be introduced to ensure consistency of the computed free Gibbs energy values. According to that, Δ*G*_(solv)_^*^ of the dissociation of a generic acid HA can be expressed as

6 $${\Delta G}_{\left(solv\right)}^{*}={\Delta G}_{PCET\left(solv\right)}^{*}+\left(G_{ET\left(water\right)}^{*}+G_{corr\left(solv\right)}^{*}\right)$$

where Δ*G*_(solv)_^*^ represents the standard Gibbs free energy change of the HA dissociation in the desired solvent, Δ*G*_PCET(solv)_^*^ corresponds to the free Gibbs energy of the proton-coupled electron-transfer process,*G*_ET(water)_^*^ is the standard-state Gibbs free energy of the pure electron-transfer reduction referenced to the aqueous SHE, and *G*_corr(solv)_^*^ is a solvent-dependent correction term that quantifies the Gibbs free energy required to transfer a proton from water into the solvent of interest under standard conditions. Taking advantage of SHE definition, it has been demonstrated that eq 6 can be rewritten as

7 $${\Delta G}_{diss\left(solv\right)}^{*}=G^{*}\left(A_{\left(solv\right)}^{-}\right)-G^{*}\left({HA}_{\left(solv\right)}\right)+G_{eff}^{*}\left(H_{\left(solv\right)}^{+}\right)$$

where the term *G*_eff_^*^(*H*_(solv)_^+^) corresponds to the effective standard-state Gibbs free energy of a proton in the target solvent. This single “proton reference” term reflects both the intrinsic proton solvation energy in water and the solvent-specific correction necessary to adjust different solvent environments. Values of *G*_eff_^*^(*H*_(solv)_^+^) have been previously computed by Busch and co-workers at the SMD/M06–2X/6–311++G­(2df,2p) level of theory, with reported values of −11.11554163 eV for DMSO and −11.08554163 eV for CH_3_CN.

Since the value of this term has been already established, only the standard-state Gibbs free energies of the deprotonated (A^–^) and protonated (HA) species in the solvent of interest need to be computed to determine Δ*G*_diss(solv)_^*^. The p*K*_a_ values can then be directly derived from Δ*G*_diss(solv)_^*^ using eq 2.

However, as demonstrated by Busch and co-workers, these values require a linear free energy scaling correction to reduce systematic deviations arising from the intrinsic limitations of the implicit solvation model employed in the calculations. For DMSO and CH_3_CN, the corrected p*K*_a_ values are derived using a linear regression where

8 $${pK}_{a}\left(DMSO\right)=0.7517{pK}_{a\;calc}\left(DMSO\right)-6.1$$

$${pK}_{a}\left({CH}_{3}CN\right)=0.8115{pK}_{a\;calc}\left({CH}_{3}CN\right)+2.3$$ 9

To enhance the accuracy of the results, the Gibbs free energies of the protonated (HA) and deprotonated (A^–^) species corresponding to BINOL-derived CPAs **1–5** were recalculated through full geometry optimization and vibrational frequency analysis at the SMD/M06–2X/6–311++G­(2df,2p) level of theory. The resulting p*K*_a_ values, corrected using the linear free energy scaling relationships, are reported in Table 3.

**Table 3:** p*K*_a_ Values in DMSO of Various BINOL-Based Chiral Phosphoric Acids Calculated Using the LFESR Approach

entry	CPAs	p*K*_a(exp)_ ^a^	p*K*_a(calc)_ isodesmic^b^	p*K*_a(calc)_ LFESR^c^	Δp*K*_a__(exp)‑(LFESR)_
1	**1**	3.37	-	2.81	0.56
2^d^	**2**	3.86	3.85	2.82	1.04
3	**3**	2.90	2.42	1.35	1.55
4	**4**	3.49	4.05	3.34	0.15
5	**5**	2.63	2.79	2.02	0.61

a Determined in DMSO by Berkessel, O’Donoghue, and co-workers using spectrophotometric methods; see ref 35.

b Calculated by the isodesmic method at the SMD/M06–2X/6–311++G­(2df,2p)//M06–2X/6–31G+(d) level of theory.

c Calculated by the LFESR approach method at the SMD/M06–2X/6–311++G­(2df,2p) level of theory.

d The computed p*K*_a(calc)_ LFESR value in CH_3_CN for compound **2** is 15.8.

From these data, it is possible to observe that the LFESR-derived p*K*_a_ values are consistently lower than those obtained by the isodesmic method, with Δp*K*_a(exp)‑(LFESR)_ ranging from 0.15 to 1.55 units. Despite this systematic underestimation, the LFESR approach is able to reproduce experimental data with reasonable accuracy, and since it does not require the use of a structurally related acid as reference, it represents an alternative to the isodesmic method.

The LFESR approach was then applied in the p*K*_a_ prediction of selected decahydroquinoxaline-based chiral phosphoric acids (Table 4). The p*K*_a_ values obtained with the LFESR approach confirm the general trends in acidity previously observed with the isodesmic method: compounds bearing EWD substituents in R^1^ or R^3^ positions consistently display lower p*K*_a_ values compared to those of chiral phosphoric acids functionalized with electron-donating substituents.

**Table 4:** Computed p*K*_a_ Values in DMSO and CH_3_CN for Chiral Phosphoric Acids Performed at the SMD/M06-2X/6-311++G­(d,p) Level of Theory According to the LFESR Approach

entry	CPA	p*K*_a(LFESR)_ DMSO	Δp*K*_a(isodesmic)‑(LFESR)_ DMSO	p*K*_a(LFESR)_ CH_3_CN	Δp*K*_a(isodesmic)‑(LFESR)_ CH_3_CN
1	**6**	2.18	+1.33	11.96	+0.40
2	**7**	1.83	+1.49	10.83	+0.80
3	**8**	3.17	+0.85	12.56	–0.01
4	**9**	2.97	+0.83	12.77	–0.52
5	**10**	4.17	+0.90	13.57	0.00
6	**11**	1.65	+1.58	11.13	–0.41
7	**12**	3.71	+0.59	13.69	+0.96
8	**13**	3.56	+1.87	13.60	+0.47
9	**14**	4.30	+1.24	13.02	+1.05
10	**15**	0.36	+0.49	9.95	–0.11
11	**16**	2.86	+0.25	12.24	+0.39
12	**17**	2.82	+2.48	12.50	+1.34
13	**18**	3.44	+1.56	12.65	+0.26
14	**19**	4.46	+0.84	12.23	+1.61
15	**20**	4.05	+1.09	13.63	–0.06
16	**21**	4.45	–1.22	12.19	–0.10
17	**22**	2.67	+0.70	12.10	+0.09
18	**23**	3.37	+1.31	13.03	+0.33
19	**24**	4.61	+1.00	13.90	+0.02
20	**25**	3.05	+0.22	12.62	–0.84
21	**26**	3.53	+0.28	12.64	–0.15
22	**27**	2.80	+0.18	11.99	+2.66
23	**28**	2.37	+1.20	12.06	–0.37
24	**29**	3.42	+0.29	12.59	–0.58
25	**30**	2.64	+0.18	12.01	–0.44
26	**31**	2.45	–0.43	11.62	–0.66
27	**32**	1.92	+0.20	11.41	–0.46
28	**33**	4.98	+1.55	14.28	+1.01
29	**34**	3.85	+0.63	12.32	+0.39

CPA **15** (R^3^ = C_6_F_5_, p*K*_a_ = 0.36 in DMSO) is confirmed as the strongest acid in the series, whereas compounds **33** (entry 28, p*K*_a_ = 4.98 in DMSO) and **14** (entry 9, p*K*_a_ = 4.30 in DMSO) represent the least acidic compounds.

Even if p*K*_a(LFESR)_ DMSO values computed with the LFESR approach are systematically lower compared to those obtained with the isodesmic method (Δp*K*_a(isodesmic)‑(LFESR)_ DMSO values are positive), the expected influence of substituents on acidity is maintained. Interestingly, compound **17** exhibits the largest p*K*_a_(DMSO) deviation between the isodesmic and LFESR methods (Δp*K*_a(isodesmic)‑(LFESR)_ DMSO = +2.48).

The same qualitative acidity order is also preserved when acidity was evaluated in CH_3_CN: compound **15** remains the most acidic one with a p*K*_a_ of 9.95, whereas CPA **33** (p*K*_a_ = 14.28) and CPA **14** (p*K*_a_ = 13.02) display the lowest acidity. In this case, Δp*K*_a(isodesmic)‑(LFESR)_ CH_3_CN values range from positive to negative but are generally smaller than those observed in DMSO.

## 3 Experimental Determination of p*K*_a_ Values for Decahydroquinoxaline-Based Chiral Phosphoric Acids

After having computationally determined the p*K*_a_ values of decahydroquinoxaline-based chiral phosphoric acids, experimental validation in DMSO was carried out. As a first attempt, the spectrophotometric method reported by Berkessel and O’Donoghue, based on phenol or naphthol derivatives as indicators, was employed to determine the p*K*_a_ of compounds **7** and **11**.^35^ However, this method proved to be unsuitable, likely due to the intrinsic spectral properties of the decahydroquinoxaline-based CPAs, which interfered with accurate absorbance measurements. Consequently, an alternative strategy based on potentiometric titration with tetrabutylammonium hydroxide was adopted.^47^

Potentiometric titrations were carried out using an AMEL potentiometer equipped with a combined electrode for pH determination (AMEL, internal solution ethanol saturated with KCl), and a 12.3 mM solution of *n*Bu_4_NH_4_OH·30H_2_O in DMSO was used as the titrant. Consecutive additions of the titrant were performed, and the potential difference was measured (see Supporting Information for further details). The method of the first derivative was employed to obtain the titration final points, and the p*K*_a_ value was obtained from the semititration point for each sample. All p*K*_a_ values were calculated with respect to the commercial sample of CPA **1**, whose p*K*_a_ in DMSO is reported to be 3.37. The titration method was first validated on CPA **1** and subsequently applied to compounds **6**, **8**, and **11**. The corresponding titration curves obtained for these CPAs are listed in Figure 1.

**Figure 1:**
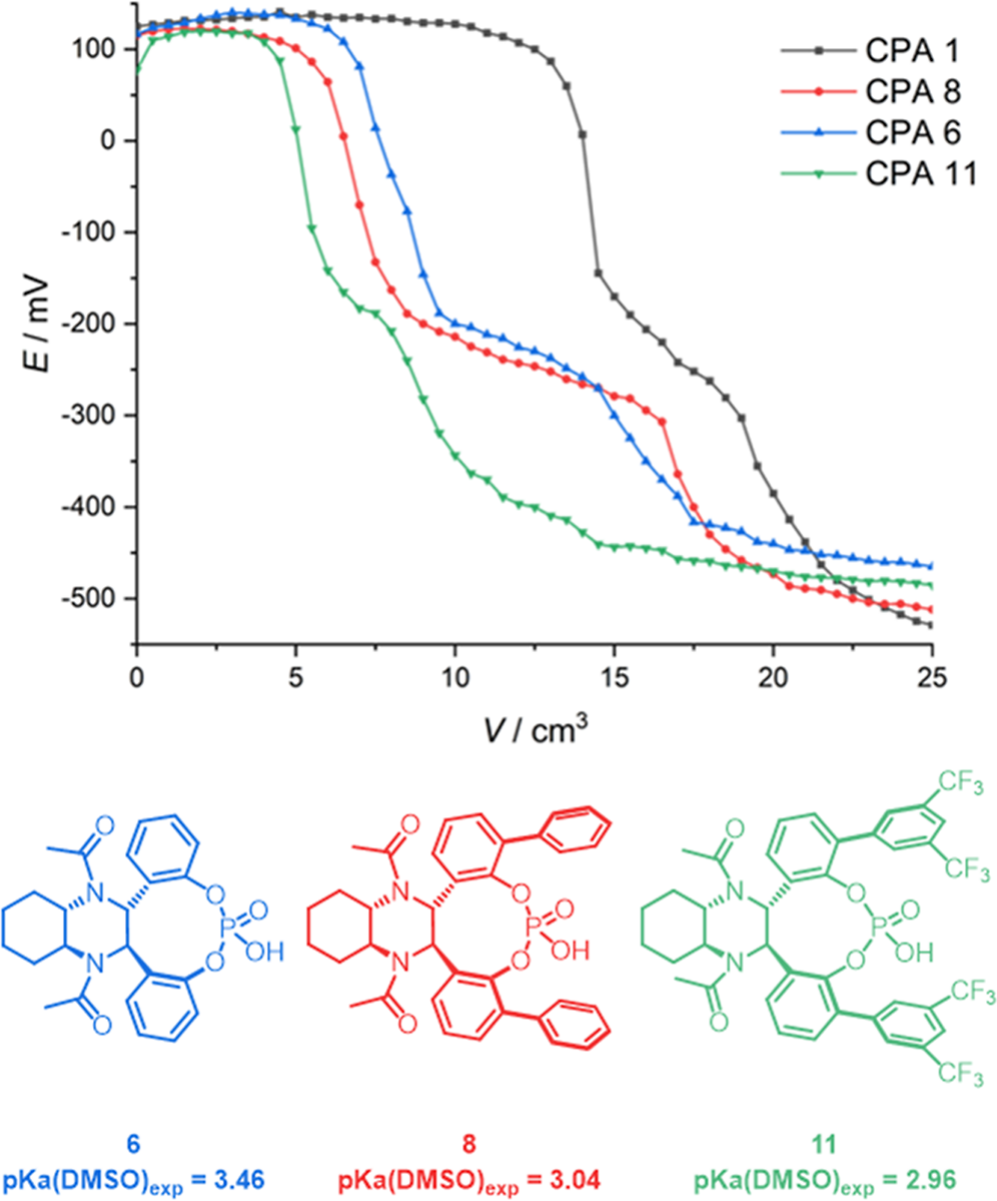
Potentiometric titration curves obtained for CPAs **1**, **6**, **8**, and **11** in DMSO and relative p*K*_a_ obtained.

As shown, all samples exhibited two distinct end points, a behavior that can be associated with the intrinsic reactivity of chiral phosphoric acids under strongly basic conditions. In the initial phase of the titration, the phosphoric acid moiety is deprotonated, leading to the formation of the corresponding phosphate salt. However, at higher base concentrations, the formation of monoester biphosphonates occurs.^48,49^ This secondary process, observed in both compounds derived from BINOL and the decahydroquinoxaline scaffold, is responsible for the extra end point observed in the titration curves. As a consequence, two p*K*_a_ values were obtained for each compound analyzed; however, for comparison with the computationally determined values, only the first p*K*_a_ value, associated with the primary deprotonation of the phosphoric acid, was considered.

The p*K*_a_ values of the decahydroquinoxaline-based chiral phosphoric acids **6**, **8**, and **11** were determined from the semiequivalence points of the corresponding titration curves and found to be 3.46, 3.04, and 2.96, respectively. These data are in good agreement with the p*K*_a_ values calculated using the isodesmic approach as the difference for CPA **6** is only 0.06 units. For compound **8**, the calculated p*K*_a_ (p*K*_a(calc)_ = 4.02) is slightly higher than the experimental value (3.04), whereas for derivative **11**, the calculated p*K*_a_ (p*K*_a(calc)_ = 2.18) is lower than the experimental value obtained.

Interestingly, phosphoric acid **8**, which shows the presence of phenyl rings at the *ortho* positions of the phosphoric acid moiety, exhibits a lower p*K*_a_ than the unsubstituted analogue **6**. This behavior highlights that acidity is not only influenced by electronic effects but also mediated by additional factors such as the presence of noncovalent interactions that further modulate the acid–base properties of the system.^8^

Having successfully determined the p*K*_a_ values of CPA **6**, **8**, and **11** in DMSO, the p*K*_a_ of compound **6** was subsequently adopted as a reference for calibrating the theoretical p*K*_a_ values calculated according to the isodesmic approach. New data obtained are listed in Scheme 5.

**Scheme 5:**
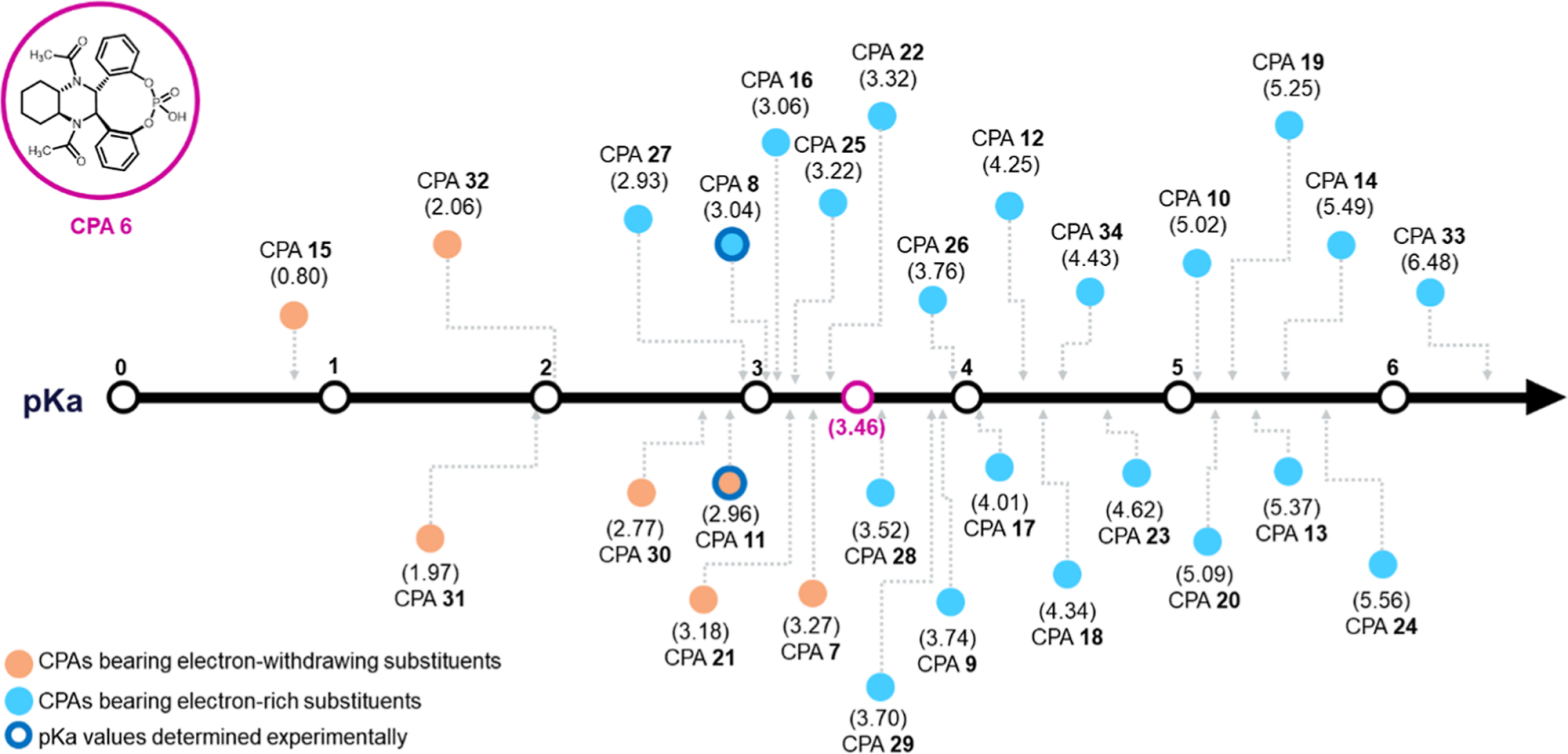
Computed p*K*_a_ Values in DMSO for Chiral Phosphoric Acids Performed at the SMD/M06-2X/6-311++G­(2df,2p) Level of Theory According to the Isodesmic Method Using p*K*_a(exp)_DMSO = 3.46 of CPA **6** as a Reference

As expected, CPAs bearing electron-withdrawing substituents show lower p*K*_a_ values compared with those featuring electron-donating groups. Interestingly, the overall agreement between experimental and calculated p*K*_a_ values is reflected by a mean absolute error (MAE) of 0.65, which indicates that, on average, the predicted p*K*_a_ values deviate from the experimental ones by less than one unit. This deviation is generally considered to be an acceptable agreement in p*K*_a_ predictions.^25^

A further comparison between data obtained from the isodesmic method and the LFESR approach was carried out. A scatter plot of corrected p*K*_a_ values calculated using the isodesmic method in DMSO *versus* those obtained from the LFESR approach in DMSO was generated (Figure 2).

**Figure 2:**
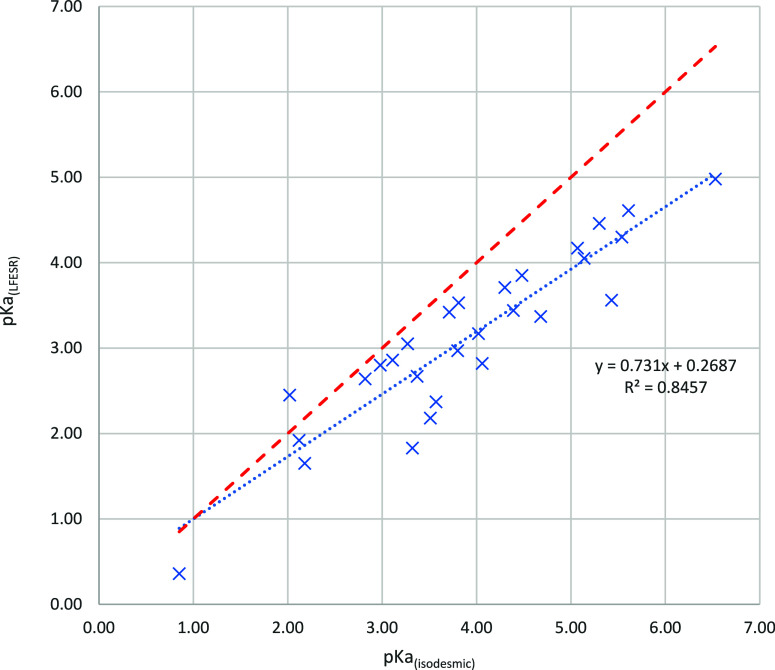
Comparison of p*K*_a_ values calculated using the corrected isodesmic method and LFESR method.

The data exhibit a strong positive correlation (RMSE = 0.90), indicating that the two approaches are broadly consistent. However, since the fitted linear regression deviates from the identity line (*y* = *x*, red line, referred to p*K*_a(calc)isodesmic_), a systematic underestimation of p*K*_a_ values by the LFESR method compared to the isodesmic method can generally be observed, particularly for CPAs with high p*K*_a_ values.

## 4 Conclusions

In conclusion, the Brønsted acidity of a novel series of C_2_ symmetrical chiral phosphoric acids featuring a decahydroquinoxaline scaffold was investigated by computational and experimental approaches. DFT calculations, performed using the isodesmic method and the linear free energy solvation relationship approach, enabled the prediction of p*K*_a_ values for 34 new CPA organocatalysts in DMSO and acetonitrile. Experimental validation by potentiometric titration on selected CPA examples confirmed the accuracy of the computational predictions, thereby supporting the reliability of the employed methodologies.

This study clearly highlights the impact of substituents on the acidity of chiral phosphoric acids, confirming that electron-withdrawing groups consistently enhance acidity, while electron-donating groups have the opposite effect. The LFESR method provides a complementary strategy to the isodesmic approach for p*K*_a_ prediction, albeit with a systematic underestimation of the p*K*_a_ values. These results not only provide a rationale for the catalytic behavior of chiral phosphoric acids but also serve as a valuable tool for the rational design of more efficient and selective CPAs to be employed in stereoselective transformations.

## Supplementary Material



## Notes

General information on solvents, reagents, and potentiometric setup; computational p*K*_a_ determination of CPAs **1–34** (according to the isodesmic method and the LFESR method); experimental p*K*_a_ determination procedures of CPAs **1**, **6**, **8**, and **11**; XYZ files of all the computed geometries (available at Dataverse UNIMI: ≪ https://doi.org/10.13130/RD_UNIMI/3TPIAE ≫) (PDF)
