# Supplementary material for: Studies on the Acidities of C2 Symmetrical Chiral Phosphoric Acids Based on a Decahydroquinoxaline Scaffold
Source: ACS Omega. 2026 Jun 17;11(25):36734–44. doi: 10.1021/acsomega.5c13291 (PMC13325088; doi:10.1021/acsomega.5c13291)
Supplement: Supplementary file 1 [file ao5c13291_si_001.pdf]

Supporting Information

for

Studies on the Acidities of C_2 Symmetrical Chiral Phosphoric Acids based on a Decahydroquinoxaline Scaffold

Margherita Gazzotti,[a] Daniele Fumagalli,[a] Valentina Pifferi,[a] Laura Raimondi,[a] and Sergio Rossi*[a]

[a] Dipartimento di Chimica, Università degli Studi di Milano, Via Golgi 19, 20133, Milano, Italy

Table of contents

1. General information 3

2. Computational pKa determination of decahydroquinoxaline-based chiral phosphoric acids 4

2.1. pKa values computed according to the isodesmic method 4

2.2. pKa values computed using the LFESR method 4

3. Experimental pKa determination of decahydroquinoxaline-based chiral phosphoric acids 10

4. References 13

1. General information

Solvents and reagents: DMSO (reagent grade, ≥ 99.9%) and tetrabutylammonium hydroxide 30-hydrate were purchased from Sigma-Aldrich and used as received.

AMEL Potentiometer: Potentiometric titrations for pKa determination were carried out using an AMEL potentiometer equipped with a combined electrode for pH determination (AMEL, internal solution ethanol saturated with KCl)

2. Computational pKa determination of decahydroquinoxaline-based chiral phosphoric acids

The initial conformational geometries were obtained through Monte Carlo conformational analysis performed with Molecular Mechanics calculations using the OPLS2005 force field[1] of the Macromodel package[2] in the Schrodinger suite.[3] For molecules exhibiting multiple conformers, the structure within 3 kcal/mol were fully optimized by DFT calculations in gas-phase using the M06-2X functional[4] with the 6-31+G(d) basis set implemented in the Gaussian package.[5]

2.1. pKa values computed according to the isodesmic method

Harmonic vibrational frequency calculations were performed at the same level of theory to confirm the nature of the stationary points and to obtain thermal corrections to the Gibbs free energy and the structure with the lowest free Gibbs energy was selected for subsequent calculations.

Single-point energy calculations were subsequently carried out on the optimized structures at the M06-2X/6-311++G(2df,2p) level of theory, including solvent effects via the SMD solvation model[6] for DMSO and CH_3CN. The previously calculated thermal corrections were then added to the electronic energies to obtain the final Gibbs free energies used for pKa determination.

The conjugate bases of each acid were generated by removing a proton from the corresponding acid structure. No conformational analysis was performed for these species; geometries were directly reoptimized using the same DFT methodology described above.

This computational protocol was applied to investigate the acidic properties of a series of BINOL-based chiral phosphoric acids **1-5** and decahydroquinoxaline-based chiral phosphoric acids **6-34**. The pKa values were computed in both DMSO and CH_3CN as solvent and results are reported in **Table S1** and **Table S4**.

All the energies calculated for the neutral chiral phosphoric acids **1-5** and the corresponding anions are reported in **Table S2** and **Table S3**. All the energies calculated for the neutral chiral phosphoric acids **6-34** and the corresponding anions are reported in **Table S5** and **Table S6**.

2.2. pKa values computed using the LFESR method

The Gibbs free energies of the protonated (HA) and deprotonated (A^-) species corresponding to BINOL-based chiral phosphoric acids **1-5** and decahydroquinoxaline-based phosphoric acids **6-34** were recalculated through full geometry optimization and vibrational frequency analysis at the SMD/M06-2X/6-311++G(2df,2p) level of theory.

For compounds **1-5** the resulting pKa values, corrected using the linear free energy scaling relationships, were computed in DMSO as solvent ($\Delta G(H^+)_{(DMSO)}$ = -11.1155416306813 eV) and results are reported in **Table S7**.

For compounds **6-34** the resulting pKa values, corrected using the linear free energy scaling relationships, were computed in both DMSO and CH_3CN ($\Delta G(H^+)_{(DMSO)}$ = -11.1155416306813 eV, ($\Delta G(H^+)_{(CH3CN)}$ = -11.0855416306813 eV) and results are reported in **Table S9**.

These values require a linear free energy scaling correction to reduce systematic deviations arising from the intrinsic limitations of the implicit solvation model employed in the calculations. For DMSO and CH_3CN, the corrected pKa values are derived using a linear regression where:

$$pKa(DMSO) = 0.7517\ pKa_{calc}(DMSO) - 6.1$$

$$pKa(CH_3CN) = 0.8115\ pKa_{calc}(CH_3CN) + 2.3$$

In a very few calculations, the convergence checks performed during the frequency step disagrees with the ones from the optimization step. However, according to Gaussian software, a geometry can be considered optimized when the Maximum Force and RMS Force are two orders of magnitude smaller than the thresholds shown, regardless of the values of the displacements. Based on that, all the geometries herein reported can be considered fully optimized.

All the energies calculated for the neutral chiral phosphoric acids **1-5** and **6-34** and the corresponding anions are reported in **Table S8** and **Table S10**.

Table S1. Computed pKa values of chiral phosphoric acids **1-5** in DMSO and CH_3CN calculated at SMD / M06-2X/6-311++G(2df,2p) // M06-2X/6-31G+(d) level of theory according to the isodesmic approach

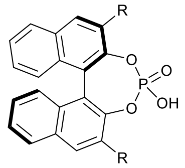

1 R = H
2 R = Ph
3 R = Br
4 R = 4-(OCH_3)C_6H_5
5 R = 3,5(CF_3)$_2C_6H_3$

Entry	CPAs	$pKa_{(exp)}$ [a]	$pKa_{(calc)}$ DMSO [b]	$pKa_{(calc)}$ DMSO [c]	$pKa_{(calc)}$ CH_3CN [c]
1	**1**	3.37	-	-	12.09
2	**2**	3.86	3.55	3.85	-
3	**3**	2.90	2.33	2.42	10.77
4	**4**	3.49	3.69	4.05	12.81
5	**5**	2.63	2.85	2.79	11.79

[a]Determined by spectrophotometric methods
[b]Calculated values at SMD(DMSO) / M06-2X/6-311++G(2df,2p) // B3LYP/6-31G+(d) by Chen, Li and co-workers[7]
[c]Calculated by SMD / M06-2X/6-311++G(2df,2p) // M06-2X/6-31G+(d)

Table S2. Complete overview of the energies calculated for the neutral chiral phosphoric acids **1-5**

	GAS PHASE (M062x 631g+ (d))		GAS PHASE (M062X6-311++G (2df,2p))		SMD (DMSO) (M062X6-311++G (2df,2p))		SMD (CH_3CN) (M062X6-311++G (2df,2p))	
CPA	Thermal correction to Free Energy (hartree)	ΔG (hartree)	ΔE (hartree)	ΔG (hartree)	ΔE (hartree)	ΔG (hartree)	ΔE (hartree)	ΔG (hartree)
1	0,2410	-1411,6560	-1412,2816	-1412,0406	-1412,2816	-1412,0406	-1412,3135	-1412,0726
2	0,3912	1.873,4393	-1.874,3478	-1.873,9566	-1874,3822	-1873,9910	-1874,3910	-1873,9998
3	0,2162	-6554,0931	-6.559,4215	-6.559,2053	-6559,4504	-6559,2343	-6559,4562	-6559,2401
4	0,4507	-2.102,3424	-2.103,3839	-2.102,9332	-2103,4225	-2102,9718	-2103,4311	-2102,9804
5	0,3850	-3.221,2666	-3.222,6234	-3.222,2384	-3222,6555	-3222,2705	-3222,6657	-3222,2807

Table S3. Complete overview of the energies calculated for the anions of chiral phosphoric acids **1-5**

	GAS PHASE (M062x 631g+ (d))		GAS PHASE (M062X6-311++G (2df,2p))		SMD (DMSO) (M062X6-311++G (2df,2p))		SMD (CH_3CN) (M062X6-311++G (2df,2p))	
CPA	Thermal correction to Free Energy (hartree)	ΔG (hartree)	ΔE (hartree)	ΔG (hartree)	ΔE (hartree)	ΔG (hartree)	ΔE (hartree)	ΔG (hartree)
1	0,2292	-1411,1605	-1411,7654	-1411,5363	-1411,7654	-1411,5363	-1411,8633	-1411,6342
2	0,3799	-1872,9462	-1.873,8355	-1873,4556	-1873,9312	-1873,55131	-1873,9399	-1873,5600
3	0,2041	-6553,6032	-6.558,9112	-6558,7071	-6559,0019	-6558,79774	-6559,0087	-6558,8045
4	0,4386	-2101,8450	-2.102,8661	-2102,4275	-2102,9704	-2102,53175	-2102,9791	-2102,5405
5	0,3741	-3220,7915	-3.222,1290	-3221,7549	-3222,2073	-3221,83314	-3222,2171	-3221,8429

Table S4. Computed pKa values of chiral phosphoric acids **6-34** in DMSO and CH_3CN calculated at SMD / M06-2X/6-311++G(2df,2p) // M06-2X/6-31G+(d) level of theory according to the isodesmic approach

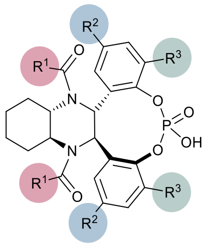

Entry	R^1	R^2	R^3	CPAs	$pKa_{(calc)}$ DMSO	$pKa_{(calc)}$ CH_3CN
1	CH_3	H	H	**6**	3.51	12.36
2	CH_3	H	Br	**7**	3.32	11.63
3	CH_3	H	Ph	**8**	4.02	12.57
4	CH_3	H	2,4,6-$(CH_3)_3C_6H_2$	**9**	3.80	12.25
5	CH_3	H	2,4,6-(iPr)$_3C_6H_2$	**10**	5.07	13.57
6	CH_3	H	3,5-$(CF_3)_2$-C_6H_3	**11**	2.18	10.72
7	CH_3	H	anthracenyl	**12**	4.30	12.73
8	CH_3	H	1-naphthyl	**13**	5.43	13.77
9	CH_3	H	2-naphthyl	**14**	5.54	14.07
10	CH_3	H	C_6F_5	**15**	0.85	9.84
11	CH_3	H	cyclohexyl	**16**	3.11	12.63
12	CH_3	H	4-(CH_2=CH-CH_2)C_6H_4	**17**	4.06	12.83
13	CH_3	H	4-(OCH_3)C_6H_4	**18**	4.39	12.91
14	CH_3	H	4-(CH_3-CH=CH)C_6H_4	**19**	5.30	13.84
15	CH_3	CH_3	Ph	**20**	5.14	13.57
16	CH_3	CH_3	3,5-$(CF_3)_2$-C_6H_3	**21**	3.23	12.09
17	H	H	H	**22**	3.37	12.19
18	H	H	Ph	**23**	4.68	13.36
19	H	H	2,4,6-(iPr)$_3$-C_6H_2	**24**	5.61	13.92
20	Ph	H	H	**25**	3.27	11.78
21	Ph	H	Ph	**26**	3.81	12.49
22	iPr	H	H	**27**	2.98	11.60
23	2,4,6-$(CH_3)_3C_6H_2$	H	H	**28**	3.57	11.69
24	2,4,6-(iPr)$_3C_6H_2$	H	H	**29**	3.71	12.01
25	3,5-$(CF_3)_2$-C_6H_3	H	H	**30**	2.82	11.57
26	CF_3	H	H	**31**	2.02	10.96
27	C_6F_5	H	H	**32**	2.12	10.95
28	1-naphthyl	H	H	**33**	6.53	15.29
29	2-naphthyl	H	H	**34**	4.48	12.71

Table S5. Complete overview of the energies calculated for the neutral chiral phosphoric acids **6-34**

	GAS PHASE (M062x 631g+ (d))		GAS PHASE (M062X6-311++G (2df,2p))		SMD (DMSO) (M062X6-311++G (2df,2p))		SMD (CH_3CN) (M062X6-311++G (2df,2p))	
CPA	Thermal correction to Free Energy (hartree)	ΔG (hartree)	ΔE (hartree)	ΔG (hartree)	ΔE (hartree)	ΔG (hartree)	ΔE (hartree)	ΔG (hartree)
6	0,4359	-1832,1156	-1.833,0806	-1.832,6446	-1833,1151	-1832,6792	-1833,1198	-1832,6839
7	0,4112	-6974,5566	-6.980,2202	-6979,8090	-6980,2577	-6979,8465	-6980,2611	-6979,8499
8	0,5872	-2293,9025	-2.295,1477	-2.294,5605	-2295,1870	-2294,5998	-2295,1948	-2294,6076
9	0,7410	-2529,5367	-2.531,0067	-2.530,2657	-2531,0432	-2530,3023	-2531,0522	-2530,3112
10	1,0686	-3000,7381	-3.002,6801	-3.001,6114	-3002,7180	-3001,6493	-3002,7299	-3001,6613
11	0,5808	-3641,7303	-3.643,4235	-3.642,8427	-3643,4601	-3642,8793	-3643,4693	-3642,8885
12	0,7658	-2908,0547	-2.909,6457	-2.908,8799	-2909,6950	-2908,9292	-2909,7068	-2908,9411
13	0,6769	-2600,9878	-2.602,4062	-2.601,7293	-2.602,4507	-2601,7738	-2.602,4588	-2601,7819
14	0,6741	-2600,9803	-2.602,3963	-2.601,7222	-2.602,4471	-2601,7730	-2.602,4559	-2601,7818
15	0,4943	-3286,0200	-3.287,5155	-3.287,0212	-3287,5661	-3287,0718	-3287,5739	-3287,0796
16	0,7260	-2300,9816	-2.302,3741	-2.301,6481	-2302,4119	-2301,6859	-2302,4194	-2301,6934
17	0,6974	-2527,1035	-2.528,5306	-2.527,8332	-2528,5728	-2527,8754	-2528,5832	-2527,8859
18	0,6470	-2522,8055	-2524,1900	-2.523,5430	-2524,2323	-2523,5854	-2524,2398	-2523,5929
19	0,6968	-2527,1128	-2.528,5387	-2.527,8419	-2528,5855	-2527,8887	-2528,5832	-2527,8859
20	0,6371	-2372,4385	-2.373,7573	-2.373,1202	-2373,7996	-2373,1625	-2373,8083	-2373,1712
21	0,6304	-3720,2636	-3.722,0308	-3.721,4004	-3722,0751	-3721,4448	-3722,0842	-3721,4538
22	0,3818	-1753,5656	-1.754,4520	-1.754,0702	-1754,4894	-1754,1076	-1754,4921	-1754,1102
23	0,5331	-2215,3552	-2.216,5221	-2.215,9890	-2216,5670	-2216,0339	-2216,5744	-2216,0413
24	1,0134	-2922,1888	-2.924,0511	-2923,0377	-2924,0904	-2923,0770	-2924,1015	-2923,0881
25	0,5363	-2215,3358	-2216,5031	-2.215,9668	-2216,5392	-2216,0029	-2216,544565	-2216,0082
26	0,6838	-2677,1184	-2.678,5641	-2.677,8803	-2678,6123	-2677,9286	-2678,6222	-2677,9384
27	0,5462	-1989,1690	-1.990,2918	-1.989,7456	-1.990,3298	-1989,7836	-1.990,3343	-1989,7881
28	0,6922	-2450,9831	-2.452,3803	-2.451,6882	-2.452,4238	-2451,7317	-2.452,4292	-2451,7371
29	1,0230	-2922,1890	-2.924,0612	-2.923,0382	-2.924,1027	-2923,0797	-2.924,1124	-2923,0894
30	0,5333	-3563,1654	-3.564,7877	-3.564,2543	-3564,8240	-3564,2906	-3564,8316	-3564,2982
31	0,3822	-2427,4311	-2.428,5463	-2.428,1641	-2.428,5788	-2428,1965	-2.428,5821	-2428,1999
32	0,4424	-3207,4928	-3.208,9140	-3.208,4715	-3.208,9450	-3208,5026	-3.208,9450	-3208,5026
33	0,6251	-2522,4293	-2.523,7719	-2.523,1467	-2.523,8200	-2523,1949	-2.523,8273	-2523,2022
34	0,6231	-2522,4258	-2.523,7651	-2.523,1421	-2.523,8102	-2523,1872	-2.523,8183	-2523,1952

Table S6. Complete overview of the energies calculated for the anions of chiral phosphoric acids **6-34**

	GAS PHASE (M062x 631g+ (d))		GAS PHASE (M062X6-311++G (2df,2p))		SMD (DMSO) (M062X6-311++G (2df,2p))		SMD (CH_3CN) (M062X6-311++G (2df,2p))	
CPA	Thermal correction to Free Energy (hartree)	ΔG (hartree)	ΔE (hartree)	ΔG (hartree)	ΔE (hartree)	ΔG (hartree)	ΔE (hartree)	ΔG (hartree)
**6**	0,4251	-1831,6351	-1.832,5799	-1832,1548	-1832,6653	-1832,2402	-1832,6700	-1832,24489
**7**	0,4000	-6974,4795	-6979,7229	-6979,3229	-6979,8080	-6979,40795	-6979,8125	-6979,41247
**8**	0,5764	-2293,4216	-2.294,6479	-2294,0715	-2294,7362	-2294,1598	-2294,7446	-2294,16819
**9**	0,7292	-2529,0510	-2.530,5015	-2529,7723	-2530,5919	-2529,8627	-2530,6017	-2529,87252
**10**	1,0584	-3001,3133	-3.002,1795	-3001,1212	-3002,2654	-3001,2070	-3002,2780	-3001,21966
**11**	0,5691	-3.641,2640	-3.642,9368	-3642,3677	-3.643,0124	-3642,4433	-3.643,0222	-3642,45309
**12**	0,7500	-2907,5709	-2.909,1383	-2908,3883	-2909,2386	-2908,4886	-2909,2513	-2908,50130
**13**	0,6638	-2600,4998	-2.601,8974	-2601,2336	-2.601,9945	-2601,3307	-2.602,0037	-2601,33988
**14**	0,6620	-2600,4952	-2.601,8916	-2601,2296	-2.601,9917	-2601,3297	-2.602,0011	-2601,33909
**15**	0,4835	-3285,5673	-3.287,0443	-3286,5608	-3287,1221	-3286,6386	-3287,1296	-3286,64614
**16**	0,7147	-2300,5053	-2301,8779	-2.301,1632	-2301,9625	-2301,2478	-2301,9685	-2301,25383
**17**	0,6868	-2526,6234	-2528,0317	-2527,3450	-2528,1221	-2527,4353	-2528,1326	-2527,44586
**18**	0,6362	-2522,3220	-2523,6876	-2523,0514	-2523,7807	-2523,14453	-2523,7889	-2523,15270
**19**	0,6851	-2526,6257	-2528,0322	-2527,3471	-2528,1309	-2527,4459	-2528,1421	-2527,4571
**20**	0,6247	-2371,9504	-2373,2491	-2372,6244	-2373,3448	-2372,72004	-2373,3543	-2372,72957
**21**	0,6199	-3719,7932	-3.721,5405	-3720,9206	-3721,6264	-3721,00645	-3721,6354	-3721,01546
**22**	0,3711	-1753,0848	-1.753,9513	-1.753,5802	-1754,0401	-1753,6690	-1754,0427	-1753,6716
**23**	0,5213	-2214,8649	-2.216,0115	-2215,4902	-2216,1138	-2215,59244	-2216,1215	-2215,60016
**24**	1,0025	-2921,7051	-2923,5495	-2922,5470	-2923,6360	-2922,63353	-2923,6483	-2922,64575
**25**	0,5226	-2214,8404	-2215,9876	-2215,4650	-2216,0870	-2215,5645	-2.216,0931	-2215,5705
**26**	0,6725	-2676,6292	-2.678,0565	-2677,3840	-2678,1615	-2677,48898	-2678,1716	-2677,49912
**27**	0,5341	-1988,6891	-1.989,7915	-1989,2574	-1.989,8799	-1989,34583	-1.989,8849	-1989,35079
**28**	0,6767	-2450,5010	-2.451,8758	-2451,1991	-2.451,9693	-2451,2926	-2.451,9763	-2451,29959
**29**	1,0115	-2921,7080	-2.923,5569	-2922,5454	-2.923,6518	-2922,6402	-2.923,6627	-2922,6512
**30**	0,5206	-3562,6893	-3.564,2900	-3563,7694	-3564,3738	-3563,85319	-3564,3816	-3563,86097
**31**	0,3711	-2426,9448	-2.428,0409	-2427,6699	-2.428,1319	-2427,76085	-2.428,1350	-2427,76397
**32**	0,4311	-3207,0190	-3.208,4200	-3207,9889	-3.208,4978	-3208,06671	-3.208,4978	-3208,06671
**33**	0,6126	-2521,9373	-2.523,2598	-2522,6473	-2.523,3619	-2522,7494	-2.523,3694	-2522,75683
**34**	0,6102	-2521,9387	-2.523,2579	-2522,6477	-2.523,3563	-2522,74616	-2.523,3657	-2522,75551

Table S7. Computed pKa values in DMSO for chiral phosphoric acids **1-5** performed at SMD / M06-2X/6-311++G(d,p) level of theory according to LFESR approach

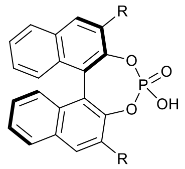

1 R = H
2 R = Ph
3 R = Br
4 R = 4-$(OCH_3)C_6H_5$
5 R = 3,5$(CF_3)_2C_6H_3$

Entry	CPAs	$pKa_{(exp)}$ [a]	$pKa_{(calc)}$ isodesmic [b]	$pKa_{(calc)}$ LFESR [c]	$\Delta pKa_{(exp)-(LFESR)}$
1	**1**	3.37	-	2.81	0.56
2	**2**	3.86	3.85	2.82	1.04
3	**3**	2.90	2.42	1.35	1.55
4	**4**	3.49	4.05	3.34	0.15
5	**5**	2.63	2.79	2.02	0.61

[a] Determined by spectrophotometric methods
[b] Calculated by isodesmic method at SMD / M06-2X/6-311++G(2df,2p) // M06-2X/6-31G+(d) level of theory
Calculated by LFESR approach method at SMD / M06-2X/6-311++G(2df,2p) level of theory.

Table S8. Complete overview of the energies calculated for chiral phosphoric acids **1-5** and the corresponding anions

Free Gibbs energies in DMSO		
CPA	ΔG_{HA} (hartree)	ΔG_{A-} (hartree)
1	-1412,0679	-1411,6293
2[a]	-1873,9910	-1873,55131
3	-6559,2343	-6558,79774
4	-2102,9718	-2102,53175
5	-3222,2705	-3221,83314

[a] ΔG_{HA} (hartree) (CH_3CN) = -1873,8691
ΔG_{A-} (hartree) (CH_3CN) = -1873,4258

Table S9. Computed pKa values in DMSO and CH_3CN for chiral phosphoric acids **6-34** performed at SMD / M06-2X/6-311++G(d,p) level of theory according to LFESR approach

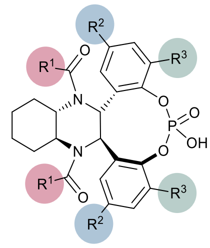

Entry	R^1	R^2	R^3	CPAs	pKa (LFESR) DMSO	ΔpKa (isodesmic)-(LFESR) DMSO	pKa (LFESR) CH_3CN	ΔpKa (isodesmic)-(LFESR) CH_3CN
1	CH_3	H	H	**6**	2.18	+1.33	11.96	+0.40
2	CH_3	H	Br	**7**	1.83	+1.49	10.83	+0.80
3	CH_3	H	Ph	**8**	3.17	+0.85	12.56	-0.01
4	CH_3	H	2,4,6-$(CH_3)_3C_6H_2$	**9**	2.97	+0.83	12.77	-0.52
5	CH_3	H	2,4,6-(*i*Pr)$_3C_6H_2$	**10**	4.17	+0.90	13.57	0.00
6	CH_3	H	3,5-$(CF_3)_2$-C_6H_3	**11**	1.65	+1.58	11.13	-0.41
7	CH_3	H	anthracenyl	**12**	3.71	+0.59	13.69	+0.96
8	CH_3	H	1-naphthyl	**13**	3.56	+1.87	13.60	+0.47
9	CH_3	H	2-naphthyl	**14**	4.30	+1.24	13.02	+1.05
10	CH_3	H	C_6F_5	**15**	0.36	+0.49	9.95	-0.11
11	CH_3	H	cyclohexyl	**16**	2.86	+0.25	12.24	+0.39
12	CH_3	H	4-(CH_2=CH-CH_2)C_6H_4	**17**	2.82	+2.48	12.50	+1.34
13	CH_3	H	4-(OCH_3)C_6H_4	**18**	3.44	+1.56	12.65	+0.26
14	CH_3	H	4-(CH_3-CH=CH)C_6H_4	**19**	4.46	+0.84	12.23	+1.61
15	CH_3	CH_3	Ph	**20**	4.05	+1.09	13.63	-0.06
16	CH_3	CH_3	3,5-$(CF_3)_2$-C_6H_3	**21**	4.45	-1.22	12.19	-0.10
17	H	H	H	**22**	2.67	+0.70	12.10	+0.09
18	H	H	Ph	**23**	3.37	+1.31	13.03	+0.33
19	H	H	2,4,6-(*i*Pr)$_3$-C_6H_2	**24**	4.61	+1.00	13.90	+0.02
20	Ph	H	H	**25**	3.05	+0.22	12.62	-0.84
21	Ph	H	Ph	**26**	3.53	+0.28	12.64	-0.15
22	*i*Pr	H	H	**27**	2.80	+0.18	11.99	+2.66
23	2,4,6-$(CH_3)_3C_6H_2$	H	H	**28**	2.37	+1.20	12.06	-0.37
24	2,4,6-(*i*Pr)$_3C_6H_2$	H	H	**29**	3.42	+0.29	12.59	-0.58
25	3,5-$(CF_3)_2$-C_6H_3	H	H	**30**	2.64	+0.18	12.01	-0.44
26	CF_3	H	H	**31**	2.45	-0.43	11.62	-0.66
27	C_6F_5	H	H	**32**	1.92	+0.20	11.41	-0.46
28	1-naphthyl	H	H	**33**	4.98	+1.55	14.28	+1.01
29	2-naphthyl	H	H	**34**	3.85	+0.63	12.32	+0.39

Table S10. Complete overview of the energies calculated for chiral phosphoric acids **2**, **6-34** and the corresponding anions

	Free Gibbs energies in DMSO		Free Gibbs energies in CH_3CN	
CPA	ΔG_{HA} (hartree)	ΔG_{A-} (hartree)	ΔG_{HA} (hartree)	ΔG_{A-} (hartree)
6	-1832,5595	-1832,1271	-1832,5646	-1832,1315
7	-6979,7277	-6979,2963	-6979,7319	-6979,3018
8	-2294,4538	-2294,0185	-2294,4615	-2294,0269
9	-2530,1454	-2529,7107	-2530,1569	-2529,7217
10	-3001,4710	-3001,0328	-3001,4835	-3001,0461
11	-3642,6629	-3642,2320	-3642,6728	-3642,2419
12	-2908,7486	-2908,3118	-2908,7603	-2908,3226
13	-2601,6085	-2601,1721	-2601,6176	-2601,1801
14	-2601,6054	-2601,1669	-2601,6151	-2601,1792
15	-3286,8720	-3286,4449	-3286,8810	-3286,4532
16	-2301,5479	-2301,1135	-2301,5540	-2301,1202
17	-2527,7154	-2527,2812	-2527,7272	-2527,2927
18	-2523,4248	-2522,9888	-2523,4329	-2522,9979
19	-2527,7300	-2527,2910	-2527,7345	-2527,3008
20	-2373,0141	-2372,5762	-2373,0230	-2372,5854
21	-3721,2292	-3720,7902	-3721,2352	-3720,80
22	-1753,9914	-1753,5576	-1753,9953	-1753,5619
23	-2215,8911	-2215,4552	-2215,8987	-2215,4628
24	-2922,9055	-2922,4661	-2922,9146	-2922,4763
25	-2215,8640	-2215,4290	-2215,8699	-2215,4351
26	-2677,7591	-2677,3227	-2677,7690	-2677,3341
27	-1989,6588	-1989,2246	-1989,6639	-1989,2307
28	-2451,5706	-2451,1376	-2451,5785	-2451,1452
29	-2922,9067	-2922,4706	-2922,9152	-2922,4805
30	-3564,0827	-3563,6489	-3564,0912	-3563,6580
31	-2428,0499	-2427,6166	-2428,0524	-2427,6203
32	-3208,3090	-3207,8773	-3208,3151	-3207,8835
33	-2523,0363	-2522,5958	-2523,0428	-2522,6036
34	-2523,0306	-2522,5934	-2523,0358	-2522,6017

3. Experimental pKa determination of decahydroquinoxaline-based chiral phosphoric acids

Potentiometric titrations for pKa determination were carried out using an AMEL potentiometer equipped with a combined electrode for pH determination (AMEL, internal solution ethanol saturated with KCl) and tetrabutylammonium hydroxide 30-hydrate as titrant (320.5 mg in 100 cm^3 of isopropanol (HPLC grade)). 20 mg of decahydroquinoxaline-based chiral phosphoric acids **6**, **8** and **11** were dissolved in 50 cm^3 of dimethyl sulfoxide (reagent grade, ≥ 99.9%) and were immediately analysed. Consecutive additions of the titrant were performed with a step of 0.5 cm^3 and the potential difference was measured.

The method of the first derivative was employed to obtain the titration final points and the pKa value was obtained from the semi-titration point for each sample. All pKa values were calculated with respect to the commercially available BINOL-based chiral phosphoric acid **1** value (pKa = 3.37).

The obtained titration curves are reported in **Figures S1-S4**.

Figure S1. Potentiometric titration of BINOL-based chiral phosphoric acid **1**

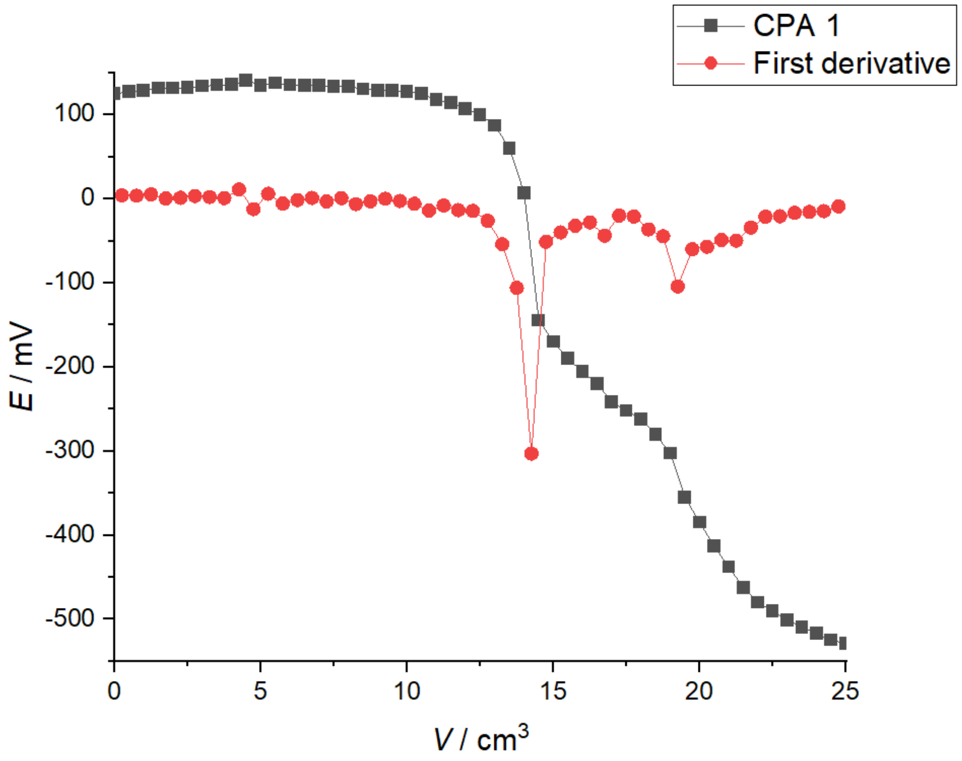

Figure S2. Potentiometric titration of decahydroquinoxaline-based chiral phosphoric acid **6**

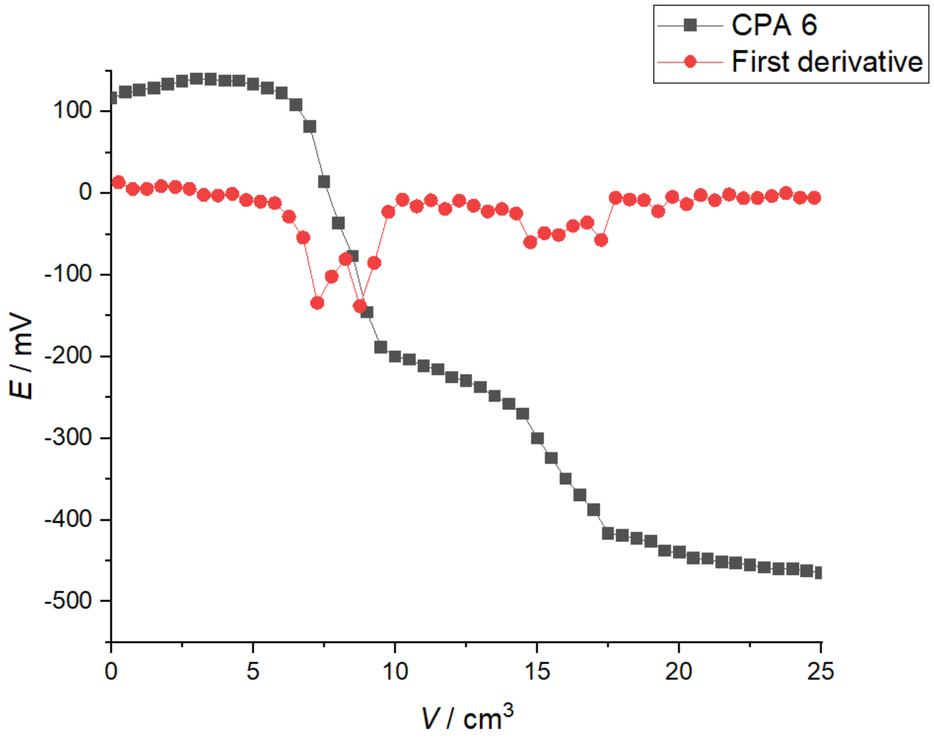

Figure S3. Potentiometric titration of decahydroquinoxaline-based chiral phosphoric acid **8**

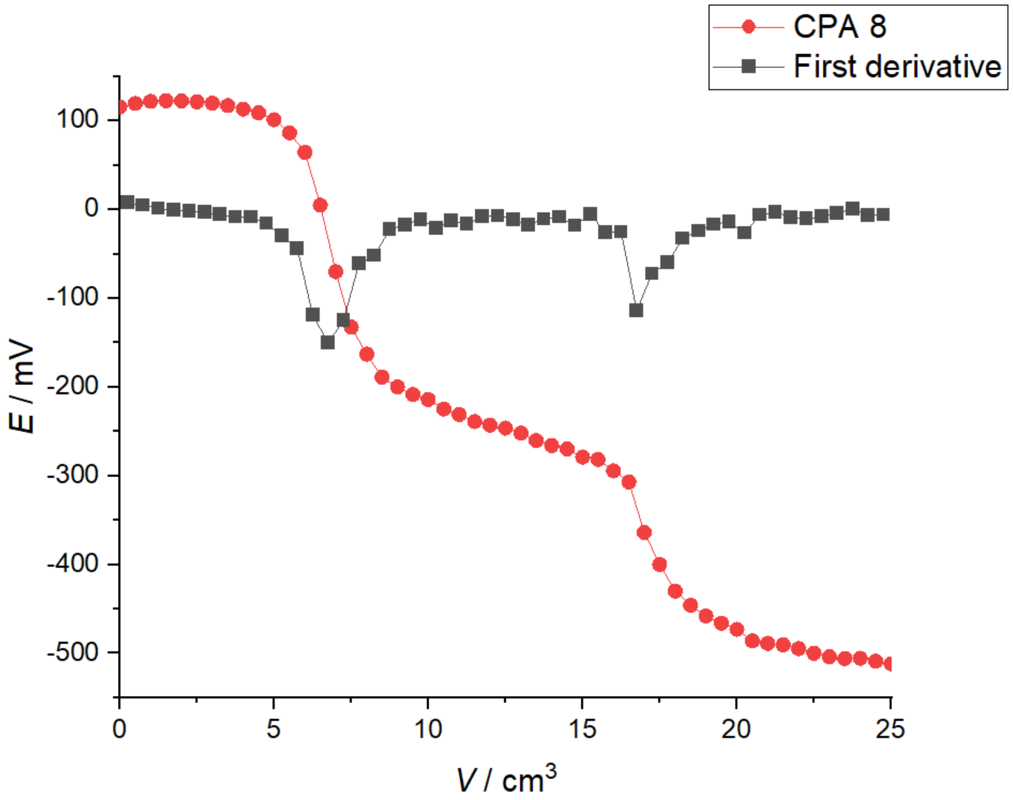

Figure S4. Potentiometric titration of decahydroquinoxaline-based chiral phosphoric acid **11**

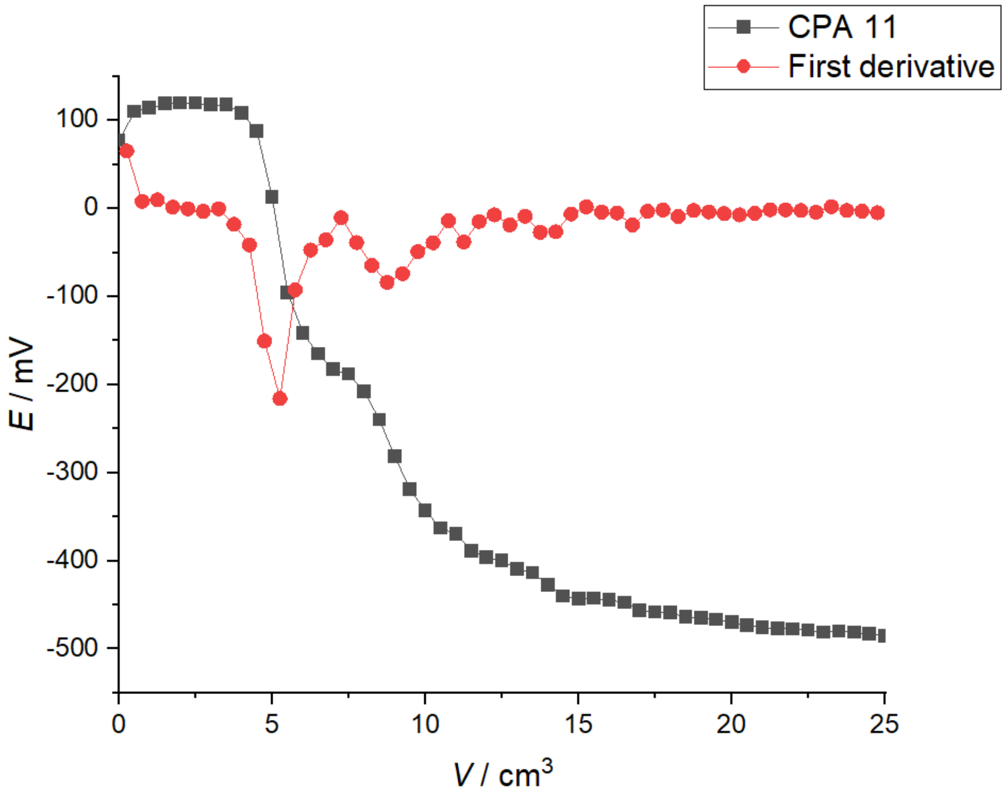

4. References

[1] M. Busch, E. Ahlberg, E. Ahlberg, K. Laasonen, *ACS Omega* **2022**, *7*, 17369–17383.

[2] J. L. Banks, H. S. Beard, Y. Cao, A. E. Cho, W. Damm, R. Farid, A. K. Felts, T. A. Halgren, D. T. Mainz, J. R. Maple, R. Murphy, D. M. Philipp, M. P. Repasky, L. Y. Zhang, B. J. Berne, R. A. Friesner, E. Gallicchio, R. M. Levy, *J Comput Chem* **2005**, *26*, 1752–1780.

[3] M. Busch, E. Ahlberg, K. Laasonen, *Phys Chem Chem Phys* **2021**, *23*, 11727–11737.

[4] Y. Zhao, D. G. Truhlar, *Theoretical Chemistry Accounts* **2007**, *120*, 215–241.

[5] Gaussian 16,Revision C.01, M. J. Frisch, G. W. Trucks, H. B. Schlegel, G. E. Scuseria, M. A. Robb, J. R. Cheeseman, G. Scalmani, V. Barone, G. A. Petersson, H. Nakatsuji, X. Li, M. Caricato, A. V. Marenich, J. Bloino, B. G. Janesko, R. Gomperts, B. Mennucci, H. P. Hratchian, J. V. Ortiz, A. F. Izmaylov, J. L. Sonnenberg, D. Williams-Young, F. Ding, F. Lipparini, F. Egidi, J. Goings, B. Peng, A. Petrone et al., Gaussian, Inc., Wallingford CT **2019**.

[6] Schrödinger Release 2024-1: MacroModel, Schrödinger, LLC, New York, NY, **2024**.

[7] C. Yang, X. S. Xue, J. L. Jin, X. Li and J. P. Cheng, *J. Org. Chem*., **2013**, *78*, 7076-7085
